# Primary Osteosarcoma of the Breast Arising in an Intraductal Papilloma

**DOI:** 10.1155/2017/5787829

**Published:** 2017-06-21

**Authors:** Khalefa Ali Alghofaily, Musab Hamoud Almushayqih, Muhannad Faleh Alanazi, Abdullah Abdulrahman Bin Salamah, Halldor Benediktsson

**Affiliations:** ^1^Radiology Department, Al Qassim University, Buraydah, Saudi Arabia; ^2^King Saud University and King Khalid University Hospital, Riyadh, Saudi Arabia; ^3^Radiology Department, Al Jouf University, Al Jawf, Saudi Arabia; ^4^King Faisal Specialist Hospital and Research Centre, Riyadh, Saudi Arabia

## Abstract

**Introduction:**

Primary osteosarcoma of the breast is extremely rare, and an osteosarcoma arising from an intraductal papilloma is exceptional.

**Case Presentation:**

A 72-year-old Saudi Arabian woman presented with a solid, bone-containing breast mass that was diagnosed as primary osteosarcoma of the breast on biopsy. She had a history of untreated intraductal papilloma. Treatment was completed with a modified mastectomy after excluding extramammary metastases. However, she subsequently developed multiple recurrent lesions at the same site.

**Conclusion:**

Primary osteogenic sarcomas of the breast are very rare. Although the main treatment is resection the optimal management remains uncertain and prognosis is poor.

## 1. Introduction

Breast cancer is the second leading cause of death in women, with invasive ductal carcinoma by far the commonest subtype. Breast sarcomas are rare neoplasms accounting for less than 1% of total breast malignancies [1]. Extraskeletal osteosarcomas have been reported throughout the body including the thyroid, kidneys, bladder, colon, testes, and penis [2]. At extraskeletal sites including the breast, osteosarcomas are thought to arise either as metaplastic differentiation of a preexisting benign or as malignant tumor or de novo from normal breast tissue. Here we present a case of osteogenic sarcoma arising in an intraductal papilloma of the breast.

## 2. Case Report

**Figure 1:**
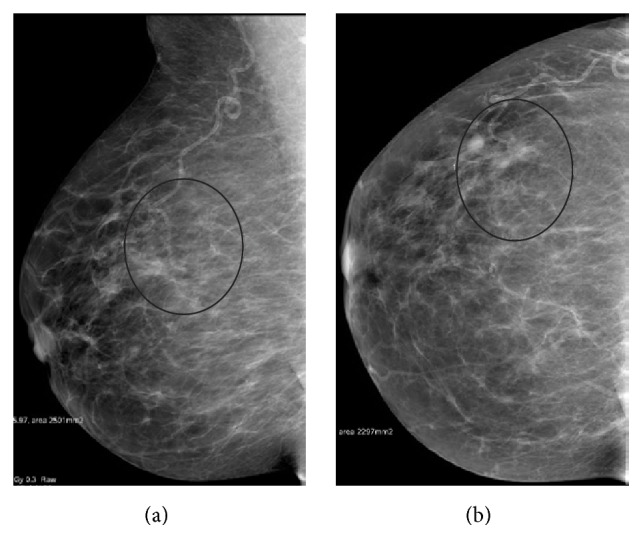
MLO (a) and CC (b) mammogram views of the right breast revealed right upper outer mid and posterior third regional clusters of microcalcifications (circle) with underlying increased density associated with partially marginated nodule; the imaging appearance is suspicious.

**Figure 2:**
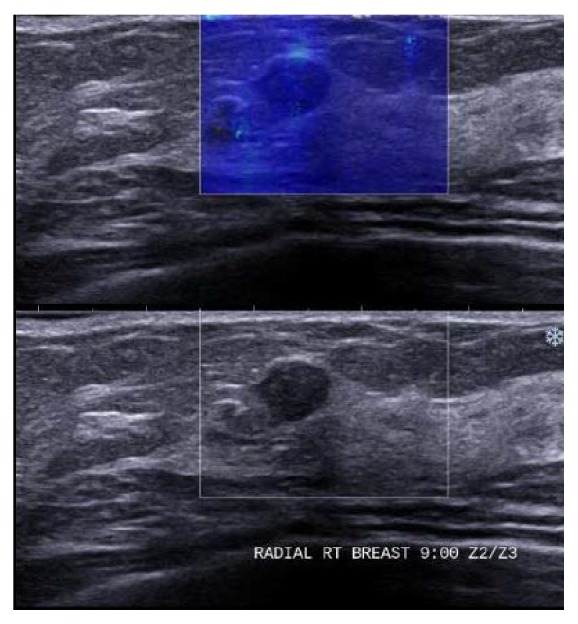
Target ultrasound corresponding to mammogram, there is partially marginated hypoechoic mass noted at 9 o'clock mid to posterior third of the right breast measuring 0.7 × 0.8 × 0.5 cm. This mass shows tubular extension from the lateral aspect and shows no internal vascularity. This mass appears relatively soft in elastography.

A 72-year-old Saudi Arabian woman had a small right breast opacity detected on a screening mammogram, which revealed right upper outer mid and posterior third regional clusters of microcalcifications with underlying increased density associated with partially marginated nodule (Figures 1(a) and 1(b)). The target ultrasound shows partially marginated hypoechoic mass with tubular extension from the lateral aspect and relatively soft in elastography (Figure 2). The lesion was asymptomatic and impalpable. Histological examination of a core biopsy revealed intraductal papilloma, and although follow-up was recommended unfortunately it was not performed.

**Figure 3:**
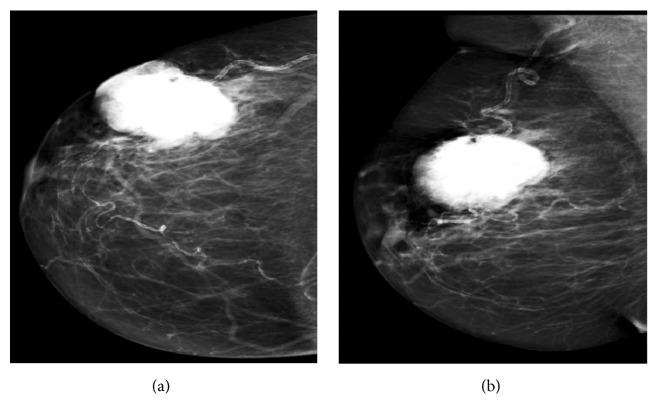
MLO (a) and CC (b) mammogram views of the right breast revealed a heavily calcified lobulated mass in the outer mid breast measuring 4.8 × 3.9 cm adjacent to a blood vessel with minimal perilesional edema.

Two years later she represented with a three-month history of a painless palpable lump in her right breast. On examination, a hard, mobile, well-circumscribed 5 cm mass occupying most of lower outer quadrant of the breast was detected. There was no nipple discharge or skin involvement and there were no palpable axillary lymph nodes. The contralateral breast, axilla, and nipple were normal. There was no history of breast trauma, radiotherapy, or hormonal therapy or a family history of breast cancer. Mammography revealed a heavily calcified, lobulated, hyperdense irregular mass in the outer mid breast. The lesion was adjacent to a blood vessel, closely resembled bone, and measured 4.8 × 3.9 cm with minimal perilesional edema. The visualized axilla was unremarkable (Figures 3(a) and 3(b)).

**Figure 4:**
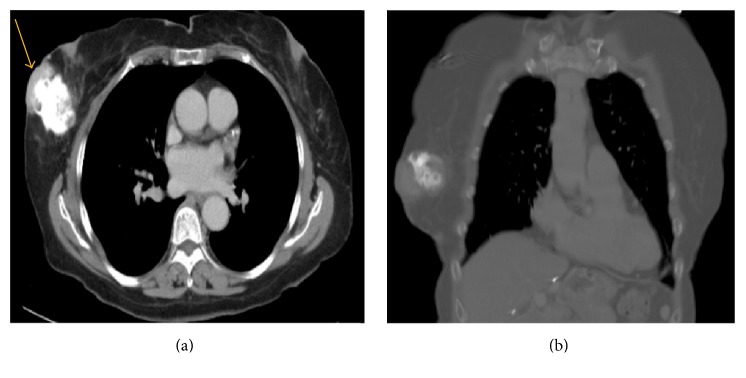
Axial (a) and coronal (b) contrast-enhanced CT to characterize the lesion and rule out metastasis. A heavily calcified right breast mass was noted measuring about 3.2 × 5.8 cm with overlying skin thickening.

Axial and coronal contrast-enhanced computerized tomography (CT) scans were ordered to better characterize the lesion and rule out metastasis. A heavily calcified right breast mass was noted in the axial plane measuring 3.2 × 5.8 cm with overlying skin thickening (Figures 4(a) and 4(b)).

The patient underwent diagnostic lumpectomy. On macroscopic examination, the mass measured 5.5 × 4.7 × 4.2 cm and was ill defined with a heterogeneous, tan, hard cut surface and extending to within 0.05 cm of the closest medial, deep, and superior margins. On histological and immunohistochemical examination, the lesion was negative CTK, CK5/6, CK7, CKLMW8/18, p63, and CK14. The lesion was diagnosed as a primary osteosarcoma of the breast.

She subsequently underwent a right modified radical mastectomy in which a 5 × 4 × 3 cm tan, firm mass in the lower outer quadrant was resected. The mass contained cystic spaces filled with blood but no further residual osteosarcoma was identified and the sentinel lymph node was negative. She did not receive any adjuvant chemotherapy but did receive close follow-up.

**Figure 5:**
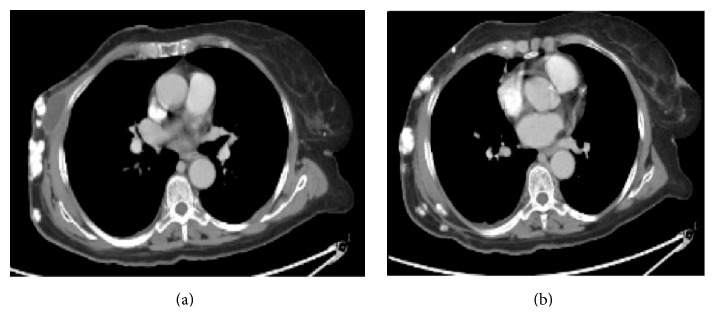
Axial contrast-enhanced postoperative CT performed after three months demonstrates postoperative changes including seroma and multiple right chest wall subcutaneous calcified nodules compatible with metastasis.

Three months after mastectomy she presented again with recurrent, multiple firm lumps in the outer lower quadrant of her right breast at the mastectomy site (Figure 5). She underwent reoperation, at which multiple, white masses were identified measuring 6 × 5.5 × 3.5 cm maximum with cystic walls underneath the previous scar. No tumor tissue was identified.

## 3. Discussion

Primary osteosarcoma of the breast (POB) is a very rare tumor, accounting for less than 1% of all breast malignancies [1]. The prognosis for patients with POB is poor, with a reported 5-year overall survival of only 38% [3].

The carcinogenetic pathway of primary osteosarcoma is poorly understood, but the leading hypotheses are that they either arise from totipotent mesenchymal cells in the breast stroma or from transformation of preexisting phyllodes tumors or fibroadenomas [3, 4]. There is a known association between a history of trauma or radiation exposure to the breast and POB [3], although neither of these factors were reported in this case.

On radiographic examination, mammary osteosarcomas usually show pathological bone formation in the breast tissue as a result of intermembranous ossification but marrow is not present [5]. The management of POB is similar to that of other sarcomas. Surgery aims to remove the lesion in its entirety with clear margins, since margin status is a major risk factor for recurrence [2, 6]. Nevertheless, mammary osteosarcomas are biologically aggressive tumors characterized by early recurrence and hematogenous metastasis, frequently to the lungs [7]. Patients may require chemotherapy when tumors are large (>5 cm) and/or high-grade, with doxorubicin, cisplatin, methotrexate, and ifosfamide showing cytotoxic effects in osteosarcomas [8]. However, administration of chemotherapy must be balanced against side effects especially in older or unfit patients, such as in this case.

## References

[1] Szajewski M., Kruszewski W. J., Ciesielski M., Śmiałek-Kusowska U., Czerepko M., Szefel J. (2014). Primary osteosarcoma of the breast: A case report. *Oncology Letters*.

[2] Thomas A. M., Nathan B. E. (1984). Primary osteosarcoma of the breast. *The British Journal of Radiology*.

[3] Silver S. A., Tavassoli F. A. (1998). Primary osteogenic sarcoma of the breast: A clinicopathologic analysis of 50 cases. *American Journal of Surgical Pathology*.

[4] Remadi S., Doussis-Anagnostopoulu I., Mac Gee W. (1995). Primary Osteosarcoma of the Breast. *Pathology - Research and Practice*.

[5] Singhal V., Chintamani, Cosgrove J. (2011). Osteogenic sarcoma of the breast arising in a cystosarcoma phyllodes: a case report and review of the literature. *Journal of Medical Case Reports*.

[6] Adem C., Reynolds C., Ingle J. N., Nascimento A. G. (2004). Primary breast sarcoma: clinicopathologic series from the Mayo Clinic and review of the literature. *British Journal of Cancer*.

[7] Kaiser U., Barth P., Duda V., Pflüger K.-H., Havemann K. (1994). Primary osteosarcoma of the breast-case report and review of the literature. *Acta Oncologica*.

[8] ESMO/European Sarcoma Network Working Group. Bone sarcomas: ESMO Clinical

